# The Situation of Young People at Home During COVID-19 Pandemic

**DOI:** 10.1007/s41255-021-00014-3

**Published:** 2021-02-19

**Authors:** Anna Lips

**Affiliations:** Department of Social and Organisational Education, University of Hildesheim, Hildesheim, Germany

**Keywords:** COVID-19 pandemic, Young people, Well-being, Home

## Abstract

The political restrictions imposed by the COVID-19 pandemic affect all population groups. However, they are of particular importance for those, whose current phase of life is mainly shaped by social life in public space – adolescents and young adults. The (at least temporary) closure of schools and universities, youth facilities, sports grounds, and pubs as well as contact restrictions changed the living conditions of young people. Life shifted mainly to the home environment and young people are obliged to deal with the people they are living with more than before. The Germany-wide survey “JuCo – Experiences and Perspectives of Young People during the COVID-19 Pandemic” asked young people about their well-being, worries and experiences during the time of lockdown. This article presents results on the situation at home and the well-being of adolescents and young adults of n = 5,520 respondents. Regression analysis is used to determine which influencing factors (e.g. money worries) affect well-being at home during the COVID-19 pandemic. Results show that different factors are influencing youth’s well being at home during the lockdown. Especially the emotional and social nature of their home environment has a very significant impact on how young respondents experience it during lockdown as well as their possibility to go outside.

## Initial Conditions for Young People in the First Weeks of the COVID-19 Pandemic

To contain the COVID-19 pandemic, in the spring of 2020, many countries introduced and enforced policies on hygiene and other protective measures. This has affected young people’s lifeworlds to a particularly high degree. They are at a stage in life when they have to detach from their parents and find their place in society. To do so, they need peers, real places to meet and the option to go outside. The closure of all public spaces from one day to the next, the restrictions on any physical contact with their peers and the message to ‘stay at home’ took away important social spaces of learning (BMFSFJ, 2020; Voigts, 2020). A message that many young people have taken very seriously (Calmbach et al., 2020; Decent Jobs for Youth, 2020; Wilmes et al., 2020).

Moreover, in this situation, young people are subject to particular risks in the fields of education, the labour market and the economy. They are, for example, more frequently affected by unemployment and earn less than older people on average. In addition, they are often in transitional situations which can be especially precarious in times of crisis (OECD, 2020). As Calmbach et al. (2020) show, the personal concerns of young people during the pandemic mainly concern these transitions, e.g. leaving school, looking for a job, moving out of the parental home, taking up an internship or a planned stay abroad (ibid.). They miss important experiences which cannot be made up.

Although young people can be seen as particularly seriously affected, and will also have to bear the long-term social and financial consequences of the COVID-19 pandemic and the political decisions made during this phase, their perspectives have not been taken into account when weighing up and making decisions in the context of the pandemic (Andresen et al., 2020a; Lips et al., 2020).

In view of this, the JuCo^1^ study, a Germany-wide online survey, asked young people aged between 15 and 30 how they saw their current situation. The survey was conducted between 15.4.2020 and 3.5.2020, in the middle of the phase when many institutions and shops in Germany were closed and very rigid contact restrictions were in place. The data provided therefore offer a targeted insight into what young people experienced during lockdown.^2^

In a period when schools, universities and child and youth welfare institutions were closed and contact with peers was largely forbidden, young people’s homes became the most important place in their lives. The fact that life was “moved indoors” presented adolescents and young adults with a variety of challenges, as far more time was spent in that context than before, and even their homes were transformed due to the changes caused by the pandemic. The young people faced the task of organising home schooling or home studying, either alone or with their parents, flatmates or partners. Some also had to restructure their everyday life due to breaks in their education and training, short-time working etc.; some had to deal with financial worries and fears about the future. In many cases, the people living with them also had to reorganise themselves from the basis of their homes. It was thus not just the case that they were spending more time together; in many respects, that time also took on a whole new quality (Andresen et al., 2020b; Bujard et al., 2020).

As a result, the discussion on contact restrictions and instructions to “stay at home” also included warnings of the potential risks to people’s relationships and health associated with spending so much time at home. Other subjects brought up in this context were the risk of an increase in domestic and sexualised violence not only of children and adolescents, but also among couples (OECD, 2020), an increase in conflict situations due to the lack of places for people to retreat to and the reorganisation of everyday life, and increasing isolation among people living alone (Geis-Thöne, 2020). There were also warnings about young people not being able to accomplish important developmental tasks (Voigts, 2020). At the same time, however, the situation could make life less hectic and, possibly, take the burden off people who feel seriously challenged by the complex demands made of them and their way of life in normal circumstances (Andresen et al., 2020a). In other words, it was particularly important to take into account the heterogeneity of young people’s lives: adolescents and young adults live in a wide range of situations, have different resources and also differ in how they deal with and perceive the situation. Groups that are considered to be particularly vulnerable, such as the homeless, the mentally ill, young refugees, care recipients and care leavers, must also be taken into account.

This article focuses on young people’s well-being in their homes. It thus starts out by examining the current state of the research to identify possible factors influencing young people’s experience in their homes, especially under the conditions of lockdown. It then goes on to shed further light on the JuCo study, the data and the methods, and provides descriptive evaluations of the dependent variables recorded in the analyses, as well as the predictors. The third step involves presenting the findings from regression analysis examining how young people experienced their home environment during lockdown. Finally, the findings are discussed along with starting points for further research.

## Determinants of Young People’s Subjective Well-being at Home

As a means of researching into how young people experience being at home, the first step will be to identify reference points in past research, to be used as a theoretical background for later analysis. In this context, it should be noted that these approaches are often formulated for adults or children, rather than explicitly for adolescents.

First, it can be stated that there are few studies overall studying subjective well-being at home as a separate dimension. Only rarely are questions asked about people’s specific experience of their home, e.g. with items such as “I am satisfied with my home” or “I have a good time at home”, or about potential factors influencing that experience (Casas et al., 2013; Klöckner & Beisenkamp, 2006; Rees & Main, 2015). In some studies, these questions are asked with reference to people’s extended living environment, such as the city district (Beisenkamp et al., 2011) or area where they live (Beisenkamp et al., 2011; Müthing & Razakowski, 2016). Available results regarding the assessment of the living environment of 9–15 year olds show that more than half of the children surveyed feel "very good" (56%) in it (Müthing & Razakowski, 2016). Until now, few questions have tended to be asked explicitly about how they experience their own home.

Instead, investigations into children’s and young people’s general well-being look into their living space or individual aspects related to it, such as how often they have moved, whether the family rents or owns their home, what condition the property is in, or whether it is overcrowded (Marcal & Foweler, 2015). Their perception and description of their living space are operationalised as predictors for their well-being or for their opportunities to develop (Coley et al., 2013). Studies on the relationship between people’s living space and their mental health and well-being have found strong links in the case of adults (cf. Clair, 2019).

So far, few findings are as yet available on the exceptional situation of the lockdown caused by the pandemic, especially in relation to adolescents and young adults. The studies which have been carried out (e.g. Calmbach et al., 2020; Decent Jobs for Youth, 2020; Langmeyer et al., 2020; Orgilés et al., 2020) only ask occasional or very specific questions about the respondents’ experiences in their home environment. The “lockdown-specific influencing factors” discussed in this article can thus not be fully explained based on the current state of the research. Moreover, in view of the limited research situation, the following exposition also includes studies from the context of various countries, even though the specifics of the lockdown may have been quite different.

Considering these restrictions, this chapter must be understood and read as reflecting theoretical frames of reference approximately. Possible factors influencing the way people experience their homes are explained following the model of previous studies on well-being and youth, theoretical discourses on youth, and initial assumptions and findings on the effects of lockdown. This article includes four types of predictor: 1. aspects that may play a predictive role in the particular context of lockdown, 2. the socio-demographic variables of sex and age, 3. factors related to the respondents’ social spaces and financial situation and 4. aspects focusing more on their social and emotional situation.

### Lockdown-specific Influencing Factors

One question this article will address is whether any factors specifically related to the COVID-19 situation and lockdown influence people’s experience of their homes; and if so, what form their influence takes. As yet, that question remains unanswered, although some possible influencing factors can be identified from a handful of studies and preliminary theoretical considerations.

One potential factor influencing people’s well-being in their home could be whether they are actually able to leave it. Initial findings on young people who have been in quarantine indicate that this experience strongly affects the respondents’ mental health (OECD, 2020; Orgilés et al., 2020). Among others, a family study by the German Youth Institute (DJI) shows that, in the parents’ opinion, young people who have access to their own garden or patio cope somewhat better with the situation during lockdown than those who do not (Langmeyer et al., 2020).

Youth studies particularly emphasise the significance of peers and social connections at this stage in life. Especially friends are considered to be particularly important for them and their well-being by young people (Andresen et al., 2019; Wolfert & Quenzel, 2019; Calmbach et al., 2020). More than half of the participant of the Shell Youth Study 2019 say that they enjoy meeting friends. The satisfaction with their circle of friends is rated quite high by the young people overall, but the 12–25 years old are less satisfied when they have contact with the majority of their friends exclusively via social media (Wolfert & Quenzel, 2019). This result is of high interest in times when the opportunities to meet in person are significantly limited. In view of this, the questions of whether the young people are in touch with others of their age, and how satisfied they are with those ties, are taken as possible predictors. This links in especially with the hypothesis that the opportunity to be in contact with people from outside their own household has multiple social functions: as well as offering them a means of escaping a space which may be perceived as confined, it can also combat loneliness and gives them a chance to discuss the current situation and face up to any challenges which arise together (Fischer et al., 2020; Geis-Thöne, 2020). Analyses by the German Socio-Economic Panel (SOEP) show that young people in particular feel lonely and miss their friends during the crisis (Entringer et al., 2020).

It is also conceivable that young people’s experience of their home is closely connected to how satisfied they are with the way they are currently living their life in the conditions of lockdown. The extensive social contact restrictions have made it impossible to carry out a large number of activities, not just in the context of school and vocational training but also in private, e.g. due to the closure of youth centres or because of sports training being cancelled. Since leisure activities were considered important by young people for their everyday satisfaction before the pandemic as Calmbach et al. (2020) show, it can be assumed that limiting them also has an impact on their well-being. At the same time, the young people and young adults may have come up with other activities (old or new) to keep them satisfied in their domestic environment. It is also conceivable that, in the conditions of lockdown and what may be perceived as a “release” from certain scheduled commitments and social obligations, some might consider themselves especially satisfied with the way they have spent their time (Andresen et al., 2020a), which would in turn affect how they experience their home.

As described in the introduction, young people and young adults are in some ways particularly profoundly affected by lockdown and its possible long-term consequences. The extent to which these young people worry about what is going on during the lockdown in Germany could be related first to their general well-being during that time and second to the well-being at home. The assumption can thus be made that if they themselves feel worried, this will be reflected in their home environment. The extent to which they feel as if attention is being paid to their worries may also be tied in with their experience at home. It is conceivable that the people living with the respondents, to whom they can talk about their worries, might have a negative effect on their well-being at home if they give the impression of not listening.

### Socio-demographic Influencing Factors

The socio-demographic factors of the respondents’ age and sex are examined as distinguishing criteria behind response patterns in almost all studies on children and young people; not only those on well-being. Findings on their experience of their home environment so far differ considerably in that respect. Of the previous studies on children’s well-being at home, the 2018 LBS Kinderbarometer, which surveyed children between the ages of 9 and 14, indicated that the well-being they experienced in their living environment decreased slightly as they aged (Müthing et al., 2018). In terms of the “family life” indicator, which partly represents well-being at home, the Children’s Worlds study also revealed slight differences related to the respondents’ age, but not to their sex (Rees & Main, 2015). Other studies show some significant differences between the sexes in questions about how respondents assess their living situation. In the study of Casas et al. (2013) girls score higher in 19 of 26 items on satisfaction, e.g. how satisfied they are with the house or flat where they live and with the people who live with them. Findings by the international Youth and Covid-19 Survey and the SINUS Youth Study also indicated that there were sex-related differences in how people viewed the situation brought about by the lockdown (Calmbach et al., 2020; Decent Jobs for Youth, 2020).

### Socio-spatial Parameters and Financial Situation

The type of housing where young people live can be seen as inextricably linked to their experience of their home – especially in times when they are subject to externally imposed social contact restrictions. Thus, for example, the risk of loneliness is named for people living alone during the COVID-19 pandemic (Mental Health Foundation, 2020).

In the context of the restrictions related to COVID-19, the DJI child study shows that situations of conflict and chaos which negatively affect the family atmosphere, and thus also the children’s experience, occur more frequently when there are several children living in a household (Langmeyer et al., 2020). Studies show that enough space in the flat or house is important for the well-being of children. If they consider their home to be too small, they are less satisfied in different areas of life, especially their living environment and their family live (Beisenkamp et al., 2011). The Shell Youth Study also shows that a cramped housing situation can increase the stress and conflict potential in a family (Wolfert & Quenzel, 2019). In times when there are few opportunities to escape their homes by going out to visit friends, child and youth work facilities, shops, cafés or pubs, this aspect may weigh especially heavily on their well-being. Several studies theorise that having enough space, their own room and a place where they are not disturbed, for example when studying or listening to music, might be a relevant factor influencing the young respondents’ home experience, opportunities to develop and education, and include questions on this subject (Klöckner & Beisenkamp, 2006; Langmeyer et al., 2020; Lopoo & London, 2016). Young people themselves also rate the importance of an undisturbed place as important for their well-being (Calmbach et al., 2020). In the context of the COVID-19 pandemic have been found to perceive primary schoolers, at least from their parents’ point of view are dealing better with the situation when they have a place of retreat (Langmeyer et al., 2020). This is likely to be at least as important for adolescents and young adults.

Almost all studies on well-being and situation in life include questions on socio-economic factors. In many of them it can be shown that the socio-economic situation is linked with the well-being of a person or family. A study of Rees and Bradshaw (2018) shows that “children living in poorer families and with parents who were less well-educated were more likely to have low life satisfaction, low happiness and high sadness” (ibid., p.40). Studies which have already been published about the effects of the COVID-19 pandemic indicate that people’s perception of the current situation and possible stressors correlate with socio-economic factors. The worse their financial situation is, the worse they consider their individual situation to be (Langmeyer et al., 2020).

It must also be assumed that a precarious financial situation can have a negative impact on the family climate (Wolfert & Quenzel, 2019) or even the general climate at home.

### Atmosphere at Home; Support and Protection

Apart from the material resources available in respondents’ homes, their relationships with co-habitants – unless they live alone – must also be considered significant (Andresen et al., 2019; Geis-Thöne, 2020; Rees & Main, 2015). With this in mind, their satisfaction with the atmosphere at home, and their specific experience of protection and support are assumed to be relevant to their home experience (cf. Beisenkamp et al., 2011; Rees & Main, 2015). The authors of the Children’s Worlds + study also highlighted the significance of high-quality relationships, pointing among other things to the relationships which children and young people have in their homes. Most of the children (8-14 years) asked in this study answered that someone in their family took care of them, although the number dwindled considerably as children grew older, from the ages of 9–14 (Andresen et al., 2019). Other studies also indicate the importance of family and social relationships for young people. Time with the family is perceived as positive by most respondents and many of the young people describe the relationship with their parents as good (Wolfert & Quenzel, 2019). Many young people name their mother as an important contact person when they have worries or problems besides friends and the partner (Berngruber et al., 2018). However, it is also shown that there are significant differences according to social class of origin—the proportion of those who report a problematic relationship increases the worse the socioeconomic situation of the family is (Wolfert & Quenzel, 2019). In the international discourse on well-being, particular importance is ascribed to feelings of protection, which is seen as fundamental to the development of children and young people (Rees & Main, 2015).

The different factors which are presented as influencing well-being at home will be examined later in this article with regard to how they actually affect young people in their homes during lockdown, based on the data from the JuCo study.

## Project Presentation

The data collected by the JuCo study between 15.4.2020 and 3.5.2020 form the basis for answering the questions about people’s experience of their home. This study asked adolescents and young adults between the ages of 15 and 30 about their experience during lockdown (Wilmes et al., 2020).

The Germany-wide online survey contains important information about how young people have experienced the situation during lockdown in different contexts. The questionnaire was designed based on the Children’s Worlds study (Rees & Main, 2015), with additional questions seen as relevant from the point of view of youth studies, and others specifically about COVID-19. Questions were asked about how satisfied the adolescents and young adults were with various aspects of their current lives, as well as relevant overall parameters and their current worries. Internationally validated instruments were adapted with the aim of designing a questionnaire that was a short as possible while also covering many aspects of life (cf. Andresen et al., 2020a).

The present article focuses especially on the young respondents’ subjective assessment of their current situation at home. Whether or not they agreed that they had a good time at home during the lockdown was thus taken as the starting point for the analysis.

Based on the data from n = 5,520 young respondents, the following questions are investigated in this article:

- How are adolescents/young adults getting on at home during lockdown?
- What factors are influencing young people’s experience at home during lockdown?

## Sampling and Social Demography

To reach as many young people as possible the snowball sampling technique was used, with a particular focus on social media. This involved sending out invitations to the survey and a digital postcard featuring the URL of the survey site via professional and private e-mail lists and, in particular, Facebook and Instagram, all within Germany. Press releases promoting the study were also created and distributed via the participating universities. Within a period of just 18 days, more than 8,500 people clicked on the survey home page. The data was cleaned, reducing the data set to n = 5,520 cases (for further details see Wilmes et al., 2020).

The respondents were 19.04 years old on average, the majority (58%) being between 15 and 18 years old. Most of the study respondents stated that they were female (65.8%), while 31.6% chose the option “male”. At the time of the survey, most of those questioned (56.6%) were at school, 18.3% were students and 11.1% were earning a living.

To examine how lockdown-specific factors, the socio-demographic characteristics of age and sex, factors related to their social spaces and financial situation and the atmosphere, support and protection they experience at home influence adolescents’ and young adults’ well-being in their homes, the characteristics and then the findings of a regression analysis are presented below.

## How are Adolescents/Young Adults Getting on at Home During Lockdown?

When the young people were asked to what extent they agreed with the statement that they were currently having a good time at home, they used the entire spectrum of five responses from “Disagree” (1) to “Fully agree” (5). Across all respondents, the mean value for this item is 3.72, with a standard deviation of SD = 1.077; there is a clearly right-skewed distribution. The majority of respondents (63.6%) agreed or fully agreed with this statement. By contrast, 4.1% of those questioned (n = 224) did not agree with it (Table 1).

Overall, the descriptive evaluation and frequency count for the above indicator of well-being at home indicate that, on average, it is relatively high among the adolescents and young adults surveyed in the JuCo study. However, the response variance also shows that it does not apply equally to all respondents.

### Lockdown-specific Influencing Factors

Influencing factors considered to be possibly specific to lockdown included being able to go outside, being in contact with peers and satisfaction with current connections. Satisfaction with how time was spent, the question of whether the young people were worried about what was happening in Germany at the time of the survey, and whether they felt attention was being paid to their worries were also included in this category.

The vast majority of respondents (96.8%) stated that they were able to go outside at the time of the survey, although it is not clear whether the 174 people who answered “no” to this question were in quarantine at the time or there were other reasons why they did not feel able to go outside, as this question was asked in a very open manner as part of the JuCo survey.

The question about how many same-age peers they were in touch with was coded in binary form for this article and thus reduced to the categories “yes, I am in touch with at least one person of the same age” and “no, I am not in touch with anyone of the same age”. 91.6% of the respondents stated that they were in touch with at least one same-age peer, but this was not the case for 8.4% of the respondents. This is a striking finding considering that the respondents are in a stage in life during which peers usually play a prominent role. When the young people were asked how satisfied they currently were with the contact they had with friends, the mean value was 4.93 on a scale from 0 (totally dissatisfied) to 10 (totally satisfied). This value is far below the satisfaction reported, for example, by the 12-year-olds from Germany in the Children's World survey with regard to the question of satisfaction with their friends. The mean value there—before the pandemic—was 8.7 (Rees et al., 2020).

Also on an 11-point scale. the respondents’ satisfaction with the way they were spending their time since the COVID-19 pandemic began was at 5.04 (SD = 2.565), with clear response variance. Thus, 8.7% of those questioned said they were very dissatisfied (1 or 2 on the scale), while 9.2% said they were very satisfied (options 9 or 10).

On a scale from 0 (disagree) to 4 (fully agree), the young people were asked to indicate whether they were worried about what was currently happening in Germany. Almost a quarter of respondents (24.6%) stated that they fully agree with this statement, a further 34.2% chose the “agree” option, around another quarter were in the middle and only a little more than 15% disagreed slightly or fully with the statement.

With respect to these and other potential worries experienced by the respondents, they were then asked whether they felt as if attention was being paid to their worries. Just under a quarter of the respondents (23.6%) stated that they did not feel as if attention was being paid to their worries. Another 21.8% selected “slightly disagree” and 29.9% were in the middle.

## Socio-spatial and Financial Factors

Socio-spatial factors include the type of housing where the young people live, the number of persons per room at home, whether they have their own room or shared, and whether they have an undisturbed space.

Table 2 shows how the respondents were distributed across the types of housing where they lived at the time of the survey. The vast majority (75.5%) said they lived with their family, 9.4% lived with their partner, 6.7% lived in shared housing and 6.3% lived alone. The smallest proportion of respondents (2.1%) lived in residential care (assisted living or a foster family).

**Table 1:** Agreement with the statement “I am currently having a good time in my home”

	Frequency	Valid percent
Disagree	224	4.1
Slightly disagree	530	9.7
Slightly agree	1244	22.7
Agree	2070	37.7
Fully agree	1421	25.9
Total	5489	100.0

**Table 2:** Type of housing where respondents live

	Frequency	Valid percent
With my family	4138	75.5
In residential care	111	2.1
Alone	346	6.3
In shared housing	368	6.7
With my partner	516	9.4
Total	5479	100.0

The respondents were also asked how many people (including them) lived in the household. Most respondents (29.2%) stated that they live in a household with four people (including them), around a quarter (24.4%) lived in a three-person household and another 16.8% stated that they lived with one other person. Only 0.5% of respondents lived in a household with more than 10 people. When asked about the number of rooms there were at home (not including the bathroom, basement, laundry room or storage room), respondents most frequently stated that there were between 3 and 6 rooms: 15.5% live in a household with 3 rooms, 17.0% in a household with 4 rooms, 16.8% in a household with 5 rooms, and 14.4% in a household with 6 rooms. Fewer than 3% of respondents stated that they lived in a household with more than 10 rooms.

10.9% of respondents live in a household where the number of habitants is higher than the number of rooms; the remaining 89.1% live in a household with as least as many rooms as there are people. The number of persons per available room is assumed to be relevant for further analysis, as it offers an insight into possible spatial confinement, similarly to the question about whether respondents have their own room and an undisturbed place of retreat.

When asked whether they had their own room, 86.3% of respondents said they had their own room, 10.7% said they had a room that they shared with someone else and 3.0% of the respondents said they did not have their own room. These values are similar to the results of the LBS Kinderbarometer 2011, where 88% of the children surveyed said they had their own room (Beisenkamp et al., 2011). Although the vast majority of the respondents of the JuCo study (90%) also stated that they had a room at home where they were undisturbed, another 7.8% stated that this was not the case and 2% could not offer a definite reply. The proportion of those who do not have an undisturbed space at home is thus lower than in the 2011 LBS children's survey, probably due to the age of the respondents, where more than 10% of the participants (9–15 years) answered that they could only "somewhat" or "not at all" agree with the statement about having an undisturbed space at home (Beisenkamp et al., 2011). However, since young people themselves rate the importance of an undisturbed place as important for their well-being (Calmbach et al., 2020), this is still to be considered problematic. Especially in a time when everybody is ment to stay home.

**Fig. 1:**
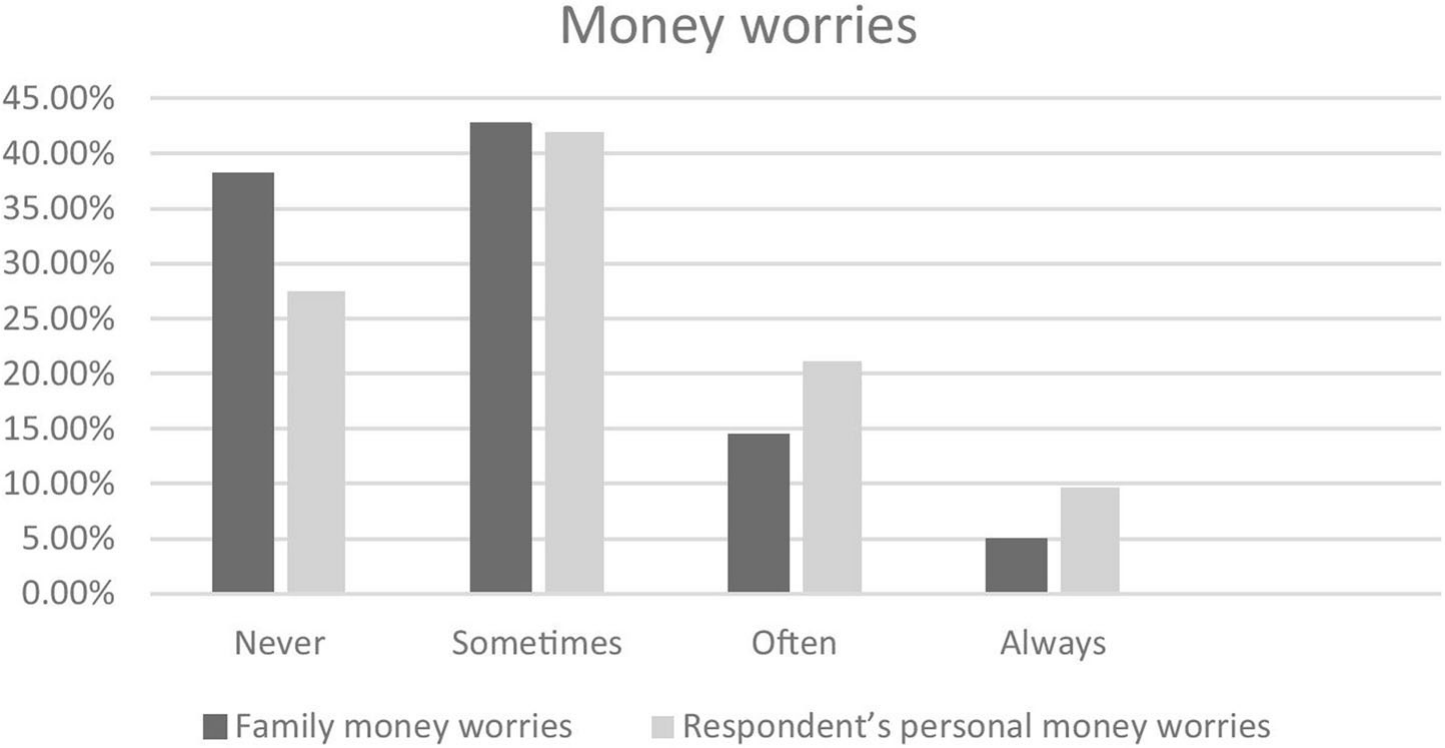
Money worries

Information on the respondents’ financial situation included how frequently they worried about their own or their family’s money. Figure 1 shows how frequently respondents worried about their own or their family’s money. 38.1% reported that they are never worried about their family money, the percentage of those who are sometimes, often or always worried about their family's financial situation is 61.9%, which is even higher than in the results of the Childrens World + study, where over 50% reported that they are at least sometimes worried about the money their family has (Andresen et al., 2019). Even more often the respondents of the JuCo study are worried about their own money (27,4% never, 41,9% sometimes, 9.6% always, 21.1% often).

### Atmosphere, Support and Protection

As described in the context of the theoretical discussion, being in high-quality relationships is assumed to be extremely relevant to the experiences of children and young people. This article records their reported satisfaction with the atmosphere at home, along with rates of agreement with the questions on whether there is currently anyone present to help with problems and the statement “I feel protected in my home”.

The young people were asked about their satisfaction with the atmosphere at home on an eleven-point scale from 0 (totally dissatisfied) to 10 (totally satisfied). The mean value reported by respondents was 6.58, indicating that they were moderately to somewhat satisfied in their home environment, with 1.5% of respondents claiming to be totally dissatisfied with the atmosphere at home and 8.6% totally satisfied.

**Fig. 2:**
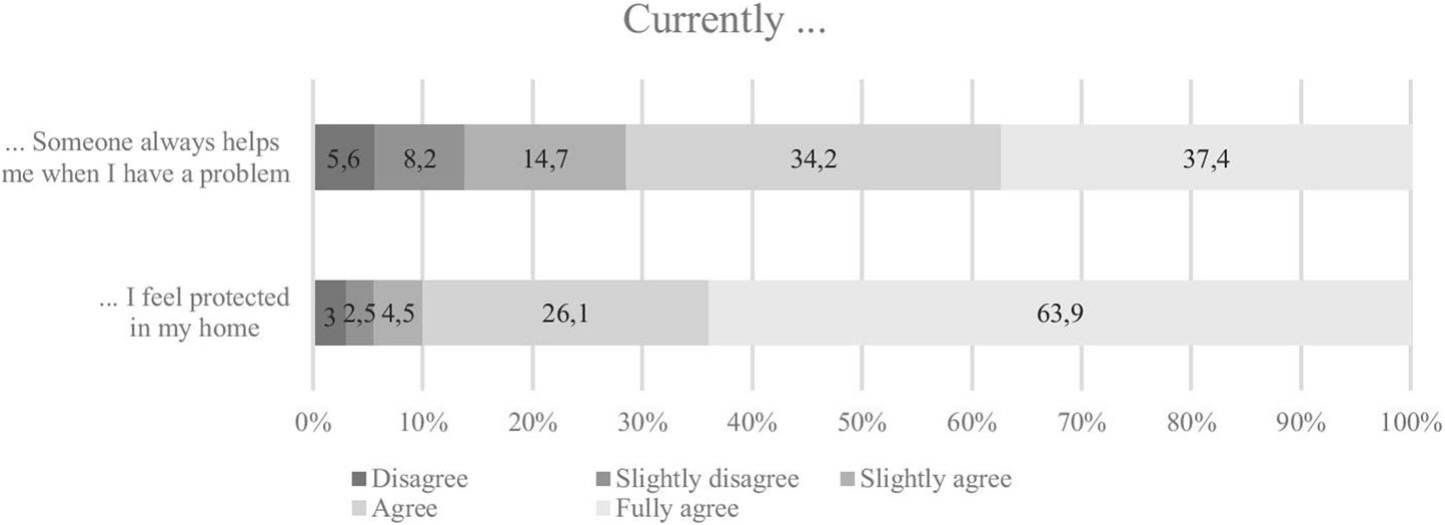
Agreement regarding protection

Figure 2 shows the agreement values for the items mentioned above in answer to the question “To what extent do you currently agree with the following statements about your home? Currently …”.

Most respondents (63.9%) felt protected in their home at the time of the survey and totally agreed with that statement. This is significantly lower than the 76% of 10-year-old respondents from Germany who fully agreed to feel safe at home in the last Children's World study (Rees et al., 2020). At the same time, however, 3% of the respondents of the JuCo survey did not agree with that statement. Most young respondents had someone to help them with problems, although those who did not agree with these statements also need to be examined here.

## Regression Analysis for Young People’s Well-being at Home During Lockdown

The following section presents the linear regression model used to investigate how potential lockdown-specific influencing factors, socio-demographic aspects, socio-spatial and financial factors, satisfaction with the atmosphere at home and young people’s experience of help and protection affect their well-being at home. To this end, four models are presented based on previous theoretical assumptions, with the predictors grouped into blocks. Both unstandardised and standardised coefficients are reported, enabling statements to be made on how strong an effect each predictor exerts.

At the time the survey was carried out, a comprehensive lockdown was in place in Germany which changed people’s everyday lives considerably and may also have affected their experiences in different contexts in various ways. The aim of the JuCo study was to record that unusual time and young people’s experiences. The first step taken was thus to input items related to the COVID-19 lockdown and the extensive social contact restrictions in place at the time of the survey into a regression model to test whether they affected people’s perception of well-being at home. Being able to go outside, the number of peers they were in contact with, their satisfaction with those connections and how their time was currently spent were all included as possible predictors, as well as questions about whether the respondents were currently worried about what was going on in Germany and whether they felt that attention was paid to their worries.

**Fig. 3:**
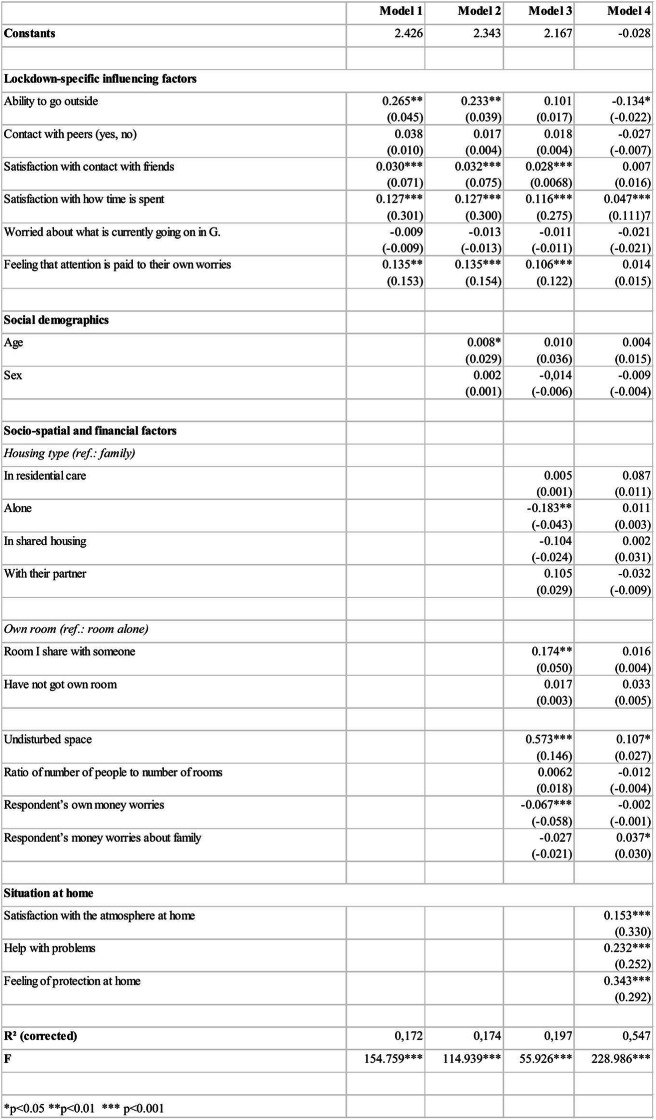
Regression models 1–4

The percentage of variance explained by Model 1 is 17.2% (corrected R^2^). In other words, 17.2% – a relatively high proportion – of the variance in people’s assessment of whether they were currently having a good time at home can be explained by the influence of the above lockdown-specific factors. Four of the six predictors – excepting the questions of whether respondents are currently worried about what is happening in Germany and whether they are currently in contact with same-age peers – have a significant influence, although to different degrees. While their satisfaction with how their time is spent and their perception of whether attention is being paid to their worries have a strong influence, their satisfaction with being in touch with friends and being able to go outside has less of an influence. Compared with the other predictors, being able to go outside or not is also revealed to have a greater influence on their agreement with the question of whether they are having a good time at home (Fig. 3).

The next step will be to set out the influence of the socio-demographic characteristics of age and sex (social demographics). It is seen that when these two variables are included, the percentage of variance explained by the model only increases slightly (corrected R^2^ = 0.174). A slightly significant positive correlation is found with the respondents’ age; that is, their level of agreement with the statement that they are having a good time at home rises slightly with age.

Picking up on the hypothesis that the nature of the living space itself may have an influence, the third step was to include socio-spatial and financial conditions in Model 3: the type of housing in which the young people live, the characteristic representing the number of persons per available room, whether or not they had their own room and whether or not they had access to an undisturbed space. Their experience of being worried about individual or family money was also added.

With regard to the types of housing in which the young respondents live, it is only those who live alone who exhibit any significant difference from those living in families. Fewer respondents living alone agreed with the statement that they were currently having a good time at home. The number of persons per room, which was coded in binary form (0 = fewer rooms than people, 1 = at least as many rooms as people) has no significant influence. One interesting finding is that people who share a room with someone agree to a significantly higher extent with the question of whether they are having a good time at home than those who indicate that they have a room to themselves. These may be the respondents who stated that they lived with their partner: they tended to agree with the statement that they were currently having a good time, with the important proviso that the effect is not significant in the presented model. In Model 3, having an undisturbed space at home had a clear influence on respondents’ well-being at home: those with access to an undisturbed space agreed significantly more frequently with the question of whether they were currently having a good time at home. Respondents in homes where the number of available rooms exceeded the number of people were more likely to agree with the statement that they were having a good time at home.

In the JuCo study, respondents’ financial situation was illustrated by the questions of how often they worried about the amount of money that was available to them personally, and to their family. Model 3 included both items to reveal any potential difference between worries about individual and family finances, partly as not all respondents lived with their families. The expected negative effect is evident. The more frequently respondents worry about their financial situation, the less likely they are to respond that they are having a good time at home – with only their individual financial worries having a significant influence. The percentage of variance explained by this model is 19.7%, i.e. 2.4% higher than Model 2.

The fourth and final step was to add characteristics to the analysis relating to respondents’ satisfaction with the atmosphere at home, the help they received with problems and whether they felt protected at home. All of the items input into Model 4 have a significant effect at a level of 0.01%, indicating clear differences. The percentage of the variance explained by the model also rises clearly (corrected R^2^ = 0.547). In Model 4, most of the other factors potentially influencing whether respondents agreed they were having a good time at home lost their significance. Moreover, the direction of the correlation is sometimes reversed in the case of those characteristics which remain significant. Whereas being able to go outside previously correlated positively, the link is now negative. Under the influence of these other factors, respondents who cannot go outside agree more frequently that they are having a good time at home. The same is true of their worries about family money. However, it should be said that the influence seems to be very small for both predictors. Their satisfaction with how their time is spent and whether they have access to an undisturbed space continue to have a significantly positive effect.

The characteristics newly added to Model 4 all have a positive effect: the higher respondents agree with these items, the more likely they are to agree with the statement that they are currently having a good time. Conversely, however, this correlation also means that respondents who agree less strongly that they have someone to talk to about problems at home, or feel protected, report a lower level of perceived well-being at home. The same is true of their satisfaction with the atmosphere at home, which also has the greatest influence in this model compared to all other characteristics and thus appears to have an especially strong influence on well-being at home.

## Conclusion and Discussion

First, based on the data from the JuCo study, it can be said that the unusual lockdown situation is indeed connected to young respondents’ well-being at home, as Model 1 shows. The characteristics included in that model alone already explain 17.2% of the variance in responses to the question of whether they agree they are having a good time at home. Adding further aspects, that value was raised to 54.7%, which can be considered extremely high.

One point which becomes clear is that the emotional and social nature of their home environment has a very significant impact on how young respondents experience it during lockdown. These are the most important factors with the strongest influence on whether the adolescents and young adults had a positive time during the first weeks of the pandemic in Germany. As Model 4 shows, the correlation is far stronger than that for factors relating to social demographics or social spaces. Whether they feel protected in their own home, the atmosphere and the support provided play a considerably more important role than factors such as whether they have their own room or what type of housing they live in. Where and how young people live thus seems to be comparatively unimportant for their feeling of well-being at home as long as there is a positive emotional basis. However, this can also be an indication of how distressing the situation can be for those who lack emotional support and a feeling of protection at home.

Therefore, it must again be specifically pointed out that, regarding the sampling used in the study, although not all questions were explicitly asked in this form, the sample is likely to be what is known as WEIRD (drawn from populations that are White, Educated, Industrialised, Rich and Democratic) (Henrich et al., 2010) and thus mainly include people with relatively high levels of socio-economic and socio-emotional stability. Conversely, this means that certain target groups (possibly those especially affected by the issue of increased vulnerability in the home environment) were not or not adequately included, and it would thus be desirable to make contact with more young people from a wide range of situations in life, so that further conclusions can be reached on necessary structures of support. The findings so far, at least, indicate that the types of support provided can be extremely important, especially in the context of relationship-based practice. Some initial reference points could be gathered from a detailed examination of the study respondents who did not agree with the statement that they were currently having a good time, as well as those who reported not feeling protected in their homes.

Taking into account these limitations to the sample of the survey, it can be said that it is a positive sign that most of the young people surveyed in the JuCo study responded that they were having a good time at home, felt protected there and received support. For most of these respondents, at least, the fear that there could be increasing conflict in people’s home environments, and that the situation might become unbearable for young people, does not appear to be borne out at the time of the survey. It would thus also be of great interest to carry out a closer examination of the possible positive effects – in both the short and long term – of spending time together and reorganising everyday life in the home environment. It will also doubtless be interesting to see what findings future studies on young people will produce with respect to family relationships, compared with previous studies.

With regard to lockdown-specific influencing factors alone, the main factors which can be seen as affecting respondents’ experience in their home environment are whether or not they can go outside and their current satisfaction with how they are spending their time. Being able to go outside is particularly relevant for those who are actually in imposed or voluntary quarantine at home. This may also be true of those who suffer from mental illness and are therefore especially affected by fears about COVID-19. It is ultimately uncertain how many people will have to go into quarantine in the coming months, and how often. In view of this study’s findings so far, and those emerging from the study on the psychological effects of quarantine on young people in Spain and Italy Italy (Orgilés et al., 2020), this unusual situation and its effects on people must be closely tracked, and strategies developed which will enable people to come out on the other side in as good a state as possible.

This article focused on respondents’ experience at home, asking the initial question of what factors influence it. At the same time, other investigations are needed to examine more precisely how young people’s well-being at home is related to their general well-being and mental health during the lockdown and beyond – whether, for instance, a high sense of well-being in their home environment plays a positive role in getting past the crisis and bearing the social consequences in the long term. It would also be important to survey the well-being at home with more and differentiated items, as the item "I have a good time at home" may only be of limited significance. It is very general and young people might associate many different things with it. The scaling of the question in the JuCo study is also to be considered problematic, as there was only one negative answer option. This maybe gave the respondent the idea that there is hardly any option “not to have a good time at home”. Perhaps young people also generally feel that—given the lockdown situation—having a not so good time might be not an option.

It could be shown that under the conditions of the lockdown, satisfaction with contact to friends and one's own leisure activities tended to decrease. Financial worries, on the other hand, were reported by more young people in the JuCo study than in other studies. These findings are a clear indicator of how much the pandemic has changed the everyday lives of young people. Having an undisturbed place at home seems to be very important for young peoples well-being even in times without a pandemic or lockdown. Perhaps it has a special role when there is hardly no possibility to go outside and when everything has to be organized at home. The analysis above (Model 3) shows the big influence this factor has on the well-being at home during the lockdown.

Altogether, it would be important and desirable to focus more strongly on the perspectives of young people themselves, and give them greater opportunity to set forth their experiences, needs and visions of the future in the discourse on COVID-19. The results emerging from the JuCo study were the first findings published in Germany to explicitly present young people’s outlooks on their situation during the lockdown. Although young people were discussed, and especially debated in the media as rule-breakers, or in their role as schoolchildren (Himmelrath, 2020; Karberg, 2020; Tagessschau.de, 2020), they themselves were asked no questions. Even when, as the discussion in the media went on, it examined the particular challenge of “being young” during the pandemic (Conrad, 2020), their perspective was still not included (Andresen et al., 2020a). It can be said that young people’s (legal) entitlement to discuss the issues affecting them on a political level, and to have a say in decisions, as laid down in the UN CRC and in Book 8 of the German Social Code (SGB), seems to have fallen out of sight both in Germany (Schröer, 2020) and internationally (Decent Jobs for Youth, 2020). During the lockdown, in particular, when many panels and committees giving a voice to young people closed down either fully or temporarily, little attention was paid to their viewpoints. A critical eye must thus be kept on this development, and suitable means found of making young people’s perspectives public and checking whether the formats designed to protect their rights to participation can stand up to a crisis.

## Data Availability

The questionnaire used can be found in the data manual oft he survey: Wilmes et al. (2020). *Datenhandbuch zur bundesweiten Studie JuCo. Online-Befragung zu Erfahrungen und Perspektiven von jungen Menschen während der Corona-Maßnahmen*. Hildesheim: Universitätsverlag.

## Footnotes

1 The JuCo study was organised by the research network on “Childhood – Youth – Family during COVID-19”. This network brings together the University of Hildesheim, Department of Social and Organisational Education and the Institute of Social Pedagogy and Adult Education at Goethe University, Frankfurt, in cooperation with Bielefeld University. So far, it has produced the nationwide JuCo study on young people’s experiences and perspectives, and the nationwide KiCo study on the experiences and perspectives of parents and their children under the COVID-19 measures.

2 The COVID-19 pandemic and related measures have seen an extended use of the term ‘lockdown’. Based on the way it is used colloquially, this article understands the term ‘lockdown’ to mean a period during which almost all shops and public facilities were closed and everywhere people stayed at home because of political orders (Klosa-Kückelhaus, 2020).

## Code Availability

Software used: IBM SPSS Statistics 26.
